# A Home-Based Exercise Program With Active Video Games for Balance, Motor Proficiency, Foot and Ankle Ability, and Intrinsic Motivation in Children With Chronic Ankle Instability: Feasibility Randomized Controlled Trial

**DOI:** 10.2196/51073

**Published:** 2023-12-20

**Authors:** Janya Chuadthong, Raweewan Lekskulchai, Claire Hiller, Amornpan Ajjimaporn

**Affiliations:** 1 Faculty of Physical Therapy, Mahidol University, Nakon Pathom, Thailand; 2 Faculty of Medicine and Health, The University of Sydney, Sydney, New South Wales, Australia; 3 College of Sports Science and Technology, Mahidol University, Salaya, Nakon Pathom, Thailand

**Keywords:** active video game, children, chronic ankle instability, motivation level, balance training, home-based exercise, video game, feasibility, ankle, instability, kinesiology, balance, exercise, exergame, exergaming

## Abstract

**Background:**

Children with chronic ankle instability (CAI) frequently experience ankle unsteadiness, causing trips, falls, and ankle sprain injuries resulting in poor quality of life. A specific exercise program focused on physical and psychological purposes in children with CAI is needed.

**Objective:**

The purpose of this study was to investigate the feasibility of a 4-week home-based exercise training program using active video games (AVGs) for balance, motor proficiency, foot and ankle ability, and intrinsic motivation in children with CAI.

**Methods:**

Sixty children with CAI (mean age 10, SD 2 years) were randomly assigned to an experimental group (AVG group; n=30) or a control group (CG; n=30). The AVG group played 2 selected video games, Catching Fish and Russian Block, while the CG received the traditional exercise program for CAI. Both programs were scheduled for 30 minutes per day, 3 times per week, for 4 weeks at home. The single-leg stance test was used to assess static balance. The walking forward on a line and standing long jump tests were used to assess motor proficiency. The Foot and Ankle Ability Measure (FAAM) and the Intrinsic Motivation Inventory questionnaire were used to assess foot and ankle ability and intrinsic motivation, respectively. Assessments were conducted at baseline and after 4 weeks.

**Results:**

In the AVG group, the single-leg stand test (eyes open; on floor and on foam conditions), the FAAM (activities of daily living subscale), and intrinsic motivation (interest/enjoyment, pressure/tension, and value/usefulness dimensions) were improved compared with the CG (all *P*<.05). Motor proficiency did not differ between the 2 groups at the end of the 4-week program (*P*=.31 for the walking forward on a line, *P*=.34 for the standing long jump test).

**Conclusions:**

A 4-week home-based exercise training program using AVGs can be beneficial and may be an effective approach for improving balance, foot and ankle ability, and enhancing positive motivation by increasing the interest/enjoyment and value/usefulness dimensions and lowering the pressure/tension dimension in children with CAI that require long-term rehabilitation sessions.

## Introduction

Chronic ankle instability (CAI) is a condition characterized by a recurring giving way of the lateral side of the ankle and usually develops after repeated ankle sprains [1]. The indicators of CAI include mechanical instability, perceived instability, and recurrent sprain, which can occur independently or in combination [1]. In children, CAI has been reported in those with a history of ankle sprains or a high BMI and in youth athletes (eg, soccer players and dancers) [2-4]. Children with CAI frequently experience ankle unsteadiness, causing trips, falls, and ankle sprain injuries [4]. To prevent the long-term detrimental impact of CAI on quality of life and activities of daily living, the feasibility of therapeutic exercise programs for children with CAI would appear warranted.

The mainstay of research to date has focused on specific exercise programs in the adult population with CAI [5-11]. For example, Seyedi et al [11] investigated the efficacy of a 4-week progressive home-based balance training program using single-leg stance, crossed-leg sway, single-leg squat, heel raise, and lunge/jump exercises in college students with functional ankle instability. The results demonstrated notable improvements in daily activity following training. Interestingly, scant research attention has focused on developing specific exercise programs in children with CAI [12,13]. To the best of the authors’ knowledge, only one study in children with foot and ankle weakness demonstrated that a 24-week high-intensity progressive resistance foot and ankle exercise program could strengthen the affected muscles and enhance the quality of life of children [14].

It is important to note that children commonly show nonadherence and lack of motivation during long-duration in-home physiotherapy training. It has been suggested that this might, in part, be due to the repetitive nature of therapeutic practices [15,16]. Therefore, much attention has recently been paid to the use of active video games (AVGs) or “exergaming” as an alternative method for therapeutic exercise in children with conditions [17,18] such as attention-deficit/hyperactivity disorder [19], type 2 diabetes [20], cerebral palsy [21], cancer [22], and developmental coordination disorder [23]. AVGs are technology-based games that track body movement or reaction to the game with instantaneous visual or audio feedback. It is proposed that this could promote physical and psychological benefits by offering the opportunity to enjoy training [18]. Furthermore, the interactive and engaging nature of the training program could result in promoting adherence and motivation through the player’s desire to complete the challenges of the game [7,24].

In children, previous studies have reported the effectiveness of AVG training on physical activity [22], gross motor skills [25,26], balance [26], daily living ability [26], voluntary motor control [27], and dynamic postural stability [28]. However, the effectiveness of AVG training in children with CAI has yet to be investigated. In a previous study at our laboratory, Chuadthong and Lekskulchai [29] compared movement patterns in children with CAI while playing 4 AVGs, including Catching Fish and Russian Block, as well as dancing and running games. The findings suggested that Catching Fish and Russian Block elicited greater specific movement patterns, such as single-leg stance, double-leg stance, and reaching direction, suggesting that these might be effective game-based rehabilitation programs for children with CAI.

Therefore, this study selected these 2 video games (ie, Catching Fish and Russian Block) from Stepmania Coilmix [30] to be used in the AVG training program due to the movement strategies of the games. We hypothesized that an AVG training program would have positive physiological and psychological outcomes in this population. More specifically, this study aimed to investigate the effectiveness of a 4-week home-based AVG exercise training program for improving balance ability, muscle strength of the foot and ankle, and intrinsic motivation in children with CAI.

## Methods

### Sample Size

This study calculated sample size estimates from a previous study [3] using G*Power (version 3.1.9.2; Universität Düsseldorf) with a Cohen *f* effect size of 0.7, an α of .05, and a power of 0.8. The calculated sample size was 54 plus 6 for 10% dropout, so a final sample size of 60 participants (30 participants for each group) was needed.

### Study Design and Participants

**Figure 1.**
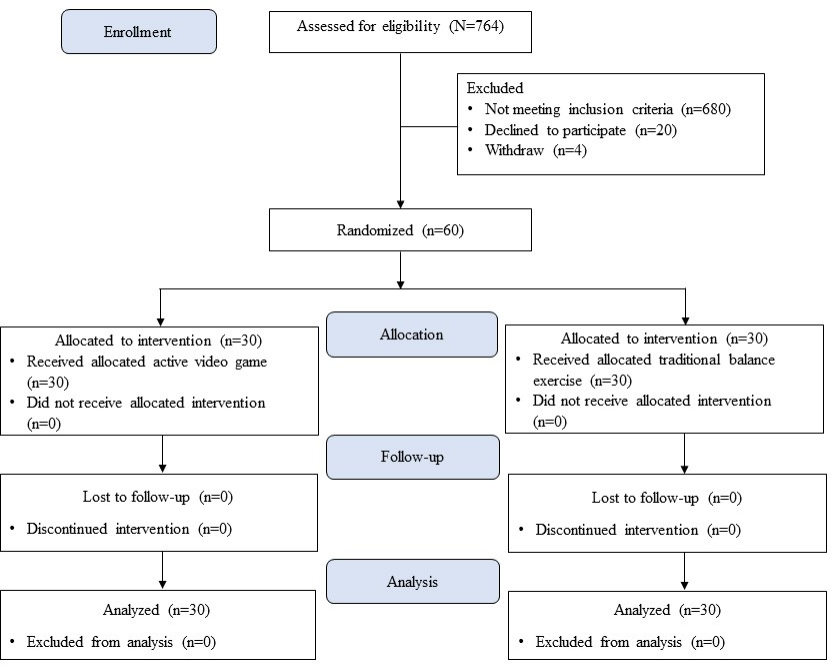
CONSORT (Consolidated Standards of Reporting Trials) flow diagram of the study participants.

This study was a single-center, assessor-blinded, randomized controlled trial. The participants were matched by age and gender and randomly assigned to either the AVG group (n=30) or the therapeutic exercise program for the control group (CG, n=30) using a computer-generated program. The CONSORT (Consolidated Standards of Reporting Trials) flow diagram of this study is shown in Figure 1. Sixty nonathletic, typically developing children with CAI aged between 7 and 12 years and with BMI between the 5th and 85th percentiles on the US Centers for Disease Control and Prevention growth chart [31] volunteered for the study. Inclusion criteria were (1) a score equal to or less than 25 on the Cumberland Ankle Instability Tool–Youth (CAITY), Thai version [32], (2) recurrent ankle sprain more than 3 months before enrollment, (3) a feeling of giving way at least twice a year, and (4) unilateral ankle sprain at least 1 year before enrollment. Participants were excluded if they had a history of either (1) ankle fracture, ankle surgery, or neurological disorder; (2) health problems such as uncontrolled seizure, asthma, severe heart disease, hearing problems, or visual problems that could not be corrected by using a lens; and (3) current participation in another rehabilitation program for the ankle joint.

### Ethical Considerations

The study conformed to the Declaration of Helsinki and was approved by the local Institutional Review Board Ethics Committee (MU-CIRB 2020/387.2311). After an explanation of the exercise protocol, testing procedures, and benefits and possible risks of the study, the informed consent form was signed by the parents or guardians and the assent form was signed by the participants.

### Experimental Procedure

The experimental group performed the AVG exercise program, whereas the CG performed the therapeutic exercise for the CAI program [33]. Both programs were scheduled for 3 sessions per week and 30 minutes per session for 4 weeks at home (a total of 12 exercise sessions). The 30-minute CG and AVG exercise programs consisted of a 5-minute active warm-up, a 20-minute main exercise, and a 5-minute cooldown. To assess compliance with the study protocol, the researchers followed up with participants once a week with a phone call. Balance ability tests and ankle instability questionnaires were evaluated at week 0 and week 4. The Intrinsic Motivation Level (IML) questionnaire was assessed each week over the course of the 4-week program [34].

### Therapeutic Exercise Program for CAI (CG)

**Figure 2.**
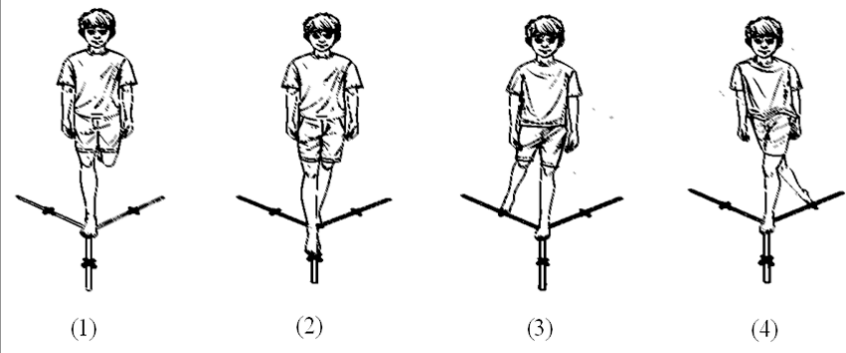
Outline of the therapeutic exercise for chronic ankle instability.

The design of the exercise program used in this study was adapted from the Star Excursion Balance Test (SEBT) [28]. Briefly, participants were asked to stand on one leg and then slowly reach with the other leg in different directions. This involved reaching in the anterior posteromedial and posterolateral directions as far as possible, touching down along the guideline, and then returning to the starting position (Figure 2). Each direction was completed as a separate trial. Participants were asked to perform 10 trials in each direction for 2 rounds with a 5-minute rest between rounds (for a total main exercise duration of around 20 minutes). Participants used their affected limbs first and then changed to the other limb. The program was designed to gradually increase the reaching distance of the leg (in centimeters) every week (by 50%, 60%, 70%, and 80% of the baseline value in weeks 1, 2, 3, and 4, respectively).

### AVG Exercise

**Figure 3.**
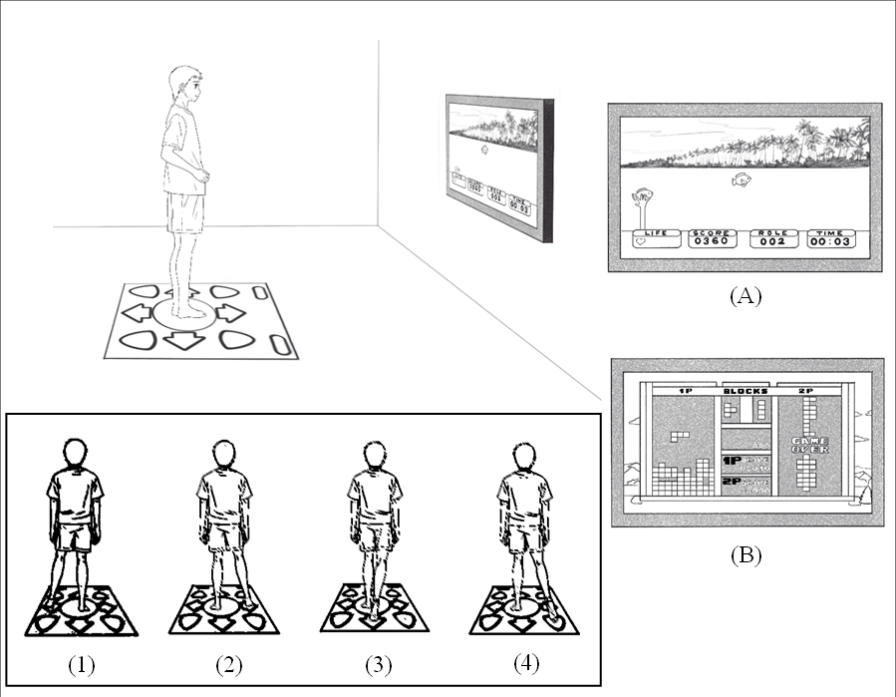
Outline of the active video games. (A) Catching Fish; (B) Russian Block.

The AVG program (Figure 3) comprised 2 selected video games (ie, Catching Fish and Russian Block) from Stepmania Coilmix, a cross-platform rhythm video game and engine with a wireless dance mat [30]. In Catching Fish, participants stood in the center of the mat before starting the game, then moved their feet either in the left or right direction in response to the game (ie, they pressed the corresponding arrows to catch the yellow or red fish). Participants had to continue playing the game as long as possible, with 4 chances to obtain a higher score. Similarly, for the Russian Block game, participants stood in the center of the mat before starting, then moved their feet in the left or right direction to change and manipulate the shape of the falling Tetromino piece shown on screen. To challenge the players, the piece will be moved faster, and it will become more difficult to correct the position. By the end of the game, the total score is shown.

The total exercise duration for the 2 games was approximately 20 minutes. If one of the games ended before 10 minutes, participants were required to start playing again from the beginning. For convenience and to promote adherence, the AVG devices were set up in the participants’ homes. The researchers provided instructions and demonstrations until the parents and children could operate the device correctly with no assistance.

### Outcome Measurements

#### Static Balance Tests

A single-leg stance test (SLST) was used to assess static balance. Participants were asked to stand on their affected limb and flex the hip and knee joints of the unaffected limb to 90 degrees for up to 30 seconds [35]. The tests were assessed in 4 conditions, including standing with eyes open (EO) or eyes closed (EC) on a stable surface (EO-floor and EC-floor) or a foam surface (EO-foam and EC-foam). The start time was when the foot was lifted off the floor. The test was stopped when any of the following occurred: (1) 30 seconds expired, (2) there were changes in the position of the weight-bearing foot, (3) any body part except the weight-bearing foot touched the floor, and (4) the participants opened their eyes during the EC trials. The test was measured 3 times. The test-retest reliability of the test was assessed and ensured using the intraclass correlation coefficient (ICC). We used the ICC (3,1) type and found that ICC was 0.793.

#### The Bruininks-Oseretsky Test of Motor Proficiency

The balance subtests (ie, walking forward on a line test and standing long jump test) of the Bruininks-Oseretsky Test of Motor Proficiency, second edition (BOT-2) [36,37] were used to assess functional balance and muscle strength. The ICC of the BOT-2 was 0.789.

For the walking forward on a line test, participants stood with feet together and hands on hips, with the preferred foot on and parallel to the line. This test was conducted one time but allowed for a second time if a participant did not achieve the maximum score of 6 correct steps. The primary investigator recorded the number of correct steps, up to 6. An incorrect step was recorded when the participant either stepped off the line, failed to keep hands on hips, or stumbled or fell.

For the standing long jump test, participants were asked to stand behind the start line, then jump forward as far as possible and try to land on their feet. This test was conducted one time; however, if the participant stumbled or fell, a second attempt was conducted. The researcher recorded the distance (in cm) from the start line to the heel.

#### Ankle Instability Questionnaire

The Foot and Ankle Ability Measure (FAAM) questionnaire is a commonly deployed diagnostic tool to assess foot and ankle ability for participation level [38,39]. It contains 2 subscales, one including 21 items on activities of daily life (FAAM-ADL) and the other 8 items for sports (FAAM-Sport). All participants rated their current level of difficulty when performing various tasks (eg, standing, walking up and down hills, and going up and down stairs for FAAM-ADL and running, jumping, and cutting or lateral movements for FAAM-Sport) with one response that most closely described their condition within the past week. If an activity was limited by something other than their foot or ankle, the question was marked not applicable (4=no difficulty, 3=slight difficulty, 2=moderate difficulty, 1=extreme difficulty, 0=unable to do, and N/A=not applicable). Percentage scores were obtained by converting item score totals ranging from 0 to 84 for the ADL subscale and from 0 to 32 for the sports subscale. A score below 90% on both subscales signifies functional ankle instability in participants. Higher scores indicate better self-reported function.

#### Intrinsic Motivation Inventory Questionnaire

The Intrinsic Motivation Inventory (IMI) is a multidimensional questionnaire used to assess the participant’s subjective perception of a target activity [34,40]. It uses a 7-point Likert scale (ranging from 1=strongly agree to 7=strongly disagree) [41]. This questionnaire assesses 5 dimensions: interest/enjoyment; perceived competence; effort/importance; pressure/tension; and value/usefulness. The mean IMI scores were calculated for each dimension. According to a previous study conducted at our laboratory, the IMI is feasible and understandable for children aged 7 years without any indications, since the questions consist of simple words and are easy to understand [29]. However, to ensure correct use, parents and children practiced under the supervision of an investigator before going home. For the first use, the investigator visited each child’s home to supervise them. Later, any problems with the use of the questionnaire were noted by the parents in the child’s logbook. Furthermore, the investigator called once a week to assess their compliance with the study protocol.

In the child’s logbook, feasibility was recorded as the rate of recruitment, retention, and adherence to the training intervention; safety was also recorded as the number of adverse events during testing or training.

### Statistical Analysis

Shapiro-Wilk tests were carried out to examine the normality of the data, and all data were found to be suitable for parametric testing. In addition, the sphericity of data was considered using the Mauchly test of sphericity; where the sphericity assumption was violated, Greenhouse-Geisser corrections were applied. Independent 2-tailed *t* tests were performed to compare the means and differences of each dependent variable at the baseline assessment. A 2-factor mixed model ANOVA was used to evaluate the effect of treatment group and time for the SLST, functional balance and strength test, and FAAM questionnaire. A repeated measure ANOVA was used for the IMI questionnaire. Any interactions between group and time were revealed using post hoc 2-tailed paired *t* tests with a Bonferroni correction to determine any differences within each group between the time points. The observed effect size was expressed as partial η squared (ηp^2^), with values of 0.1-0.29, 0.3-0.49, and >0.50 representing a small, medium, and large effect size, respectively [42]. The change score was calculated from the difference between data from week 4 minus data from week 0 using independent 2-tailed *t* tests. A *P*<.05 was considered statistically significant.

## Results

Demographic characteristics and screening tests of participants are displayed in Table 1. Independent 2-tailed *t* tests showed no significant differences between the AVG and the CG at week 0 at the *P*>.05 level.

**Table 1.** Baseline characteristics of the active video game (AVG) group and the control group (CG), which received therapeutic exercise for chronic ankle instability *(*n=30 for each group). Data were analyzed with an independent 2-tailed *t *test. The significance level was set at *P*<.05.

Characteristics		AVG group	CG	*P* value
**Gender, n**				.43
	Boy	14	11	
	Girl	16	19	
Age (years), mean (SD)		10 (2)	10 (2)	.51
Weight (kilograms), mean (SD)		32.8 (9.6)	34.0 (11.1)	.68
Height (centimeters), mean (SD)		135.2 (11.7)	136.7 (11.9)	.62
**Side of chronic ankle instability, n**				.43
	Right	20	17	
	Left	10	13	

For the SLST, the mixed model ANOVA tests revealed significant interactions between groups for EO-floor (*F*_1,58_=7.11*; P*=.004; ηp^2^=0.02) and EO-foam (*F*_1,58_=7.80; *P=.*002; ηp^2^=0.03). Further post hoc analysis revealed that the AVG group had higher values than the CG for EO-floor (*P*=.03) and EO-foam (*P*=.01) at week 4. In addition, the differences in change score between groups indicated that the AVGs elicited a change in EO-floor (95% CI=−8.01 to 0.71; *P*=.04) and EO-foam (95% CI=−7.47 to 0.05; *P*=.03) over the CG at week 4. However, there were no changes in EC-floor between time and groups for either group (*P*=.44) (Table 2).

**Table 2.** Observations from the single-leg stance test (SLST) with eyes open (EO) and eyes closed (EC) on floor and foam; the balance and strength subtests of the Bruininks-Oseretsky Test (BOT-2), including the walking forward on a line test and standing long jump test; and the Foot and Ankle Ability Measure (FAAM) for activities of daily life (ADLs) and sports at baseline (week 0) and week 4 in the active video game (AVG) exercise group and control group (CG), which received therapeutic exercise for chronic ankle instability (n=30 for each group). The significance level was *P*<.05.

Variables		AVG group			CG		
		Week 0	Week 4	Change score	Week 0	Week 4	Change score
**SLST (seconds), mean (SE)**							
	EO-floor	24.6 (1.2)	25.3 (1.1)	0.7 (1.3)	22.2 (1.4)	19.3 (1.6)^a^	−2.9 (1.7)^b^
	EC-floor	8.8 (0.9)	9.9 (1.1)	1.1 (1.0)	8.7 (1.0)	8.2 (1.1)	−0.5 (1.0)
	EO-foam	15.9 (1.4)	18.0 (1.4)	2.1 (1.3)	13.1 (1.4)	11.5 (1.3)^a^	−1.7 (1.4)^b^
	EC-foam	3.1 (0.2)	3.6 (0.3)	0.5 (0.3)	3.1 (0.2)	3.3 (0.2)	0.2 (0.2)
**BOT-2**							
	Walking forward on a line (steps), mean (SE)	5 (0)	6 (0)	0.7 (1.7)	5 (0)	6 (0)	0.4 (1.4)
	Standing long jump (centimeters), mean (SE)	107.9 (4.9)	108.5 (4.8)	0.6 (3.2)	103.6 (4.4)	106.8 (2.9)	3.2 (3.1)
**FAAM (%), mean (SE)**							
	FAAM-ADL	87.2 (1.3)	91.3 (1.1)^c^	4.3 (0.9)	82.2 (2.1)	84.1 (2.1)^a^	2.0 (1.1)^b^
	FAAM-Sports	89.6 (1.9)	91.5 (1.4)	1.9 (2.1)	83.7 (3.0)	87.1 (2.4)	3.3 (2.8)

a Significant difference between the AVG group and the CG at week 4 using mixed model ANOVA.

b Significant difference between the AVG group and the CG for change score using independent 2-tailed *t* test.

c Significant difference between week 0 and week 4 using mixed model ANOVA.

Regarding the BOT-2, including the functional balance and strength test, there were no significant interactions between time and group on the walking forward on a line (*P*=.31) and the standing long jump test (*P*=.34) in both groups (Table 2).

For the FAAM-ADL, the mixed model ANOVA tests revealed significant interactions between groups (*F*_1,58_=6.77; *P=.01*; ηp^2^=0.56) where the FAAM-ADL values for the AVG group were greater than those for the CG at week 4 (*P*=0.01). Also, significant interactions between times were found (*F*_1,58_=19.03; *P*<.0001; ηp^2^=0.25) in the AVG group, but not in the CG. The AVG group increased mean percentage on the FAAM-ADL from week 0 to week 4 (*P*<.001). Moreover, the differences in change score between groups indicated that the AVG group showed a change in percentage on the FAAM-ADL (95% CI−5.16 to 0.445; *P*=.04) over the CG at week 4. However, there were no changes in FAAM-Sports between time and group for either group (*P*=.69) (Table 2).

**Figure 4.**
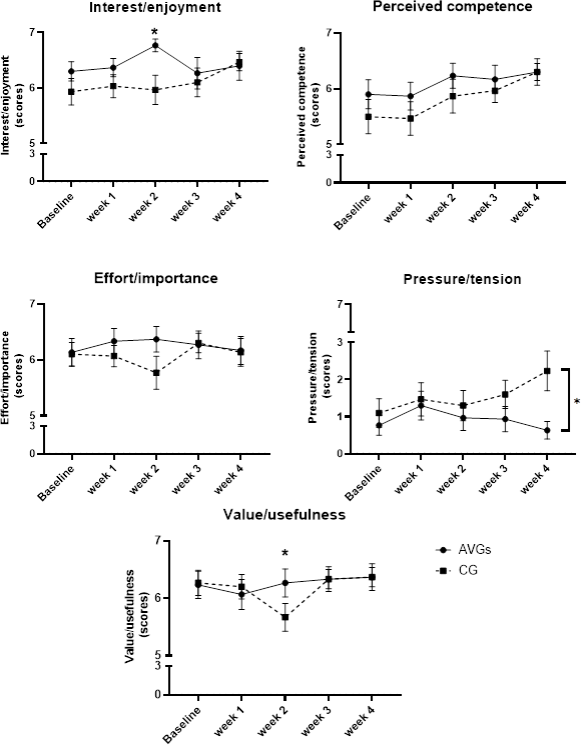
Observations from the intrinsic motivation inventory questionnaire at baseline, week 1, week 2, week 3, and week 4 in the active video game (AVG; n=30) exercise group and the control group (CG; n=30), who received therapeutic exercise. The data are shown as the mean (SEM). *P<.05; repeated measures ANOVA.

For the IMI, A 2-way repeated-measures ANOVA determined an effect of group for the interest/enjoyment (*F*_4,116_=2.59; *P*=.04; ηp²=0.08), value/usefulness (*F*_4,116_=2.71 *P*=.03; ηp²=0.09), and pressure/tension (*F*_4,116_=2.50, *P*=.04, ηp²=0.08) dimensions. Post hoc pairwise comparisons using Bonferroni correction showed a significantly greater score for interest/enjoyment and value/usefulness on the IMI in the AVG group compared to the CG (AVG group vs CG: 7, SD 0 vs 6, SD 0 for interest/enjoyment, *P*<.001 and 6, SD 0 vs 5, SD 0 for value/usefulness, *P*=.005) at week 2. Additionally, at week 4, the AVG group had a lower score for pressure/tension on the IMI than the CG (AVG group vs CG: 1, SD 0 vs 2, SD 0; *P*<.001). Significant interactions for time were found in the CG (*F*_4,116_=4.39; *P=*.002; ηp^2^=0.17) but not in the AVG group. The CG had increased perceived competence scores from baseline to week 4 (baseline vs week 4: 5, SD 0 vs 6, SD 0; *P*=.005) and week 1 to week 4 (week 1 vs week 4: 5, SD 0 vs 6, SD 0; *P*<.001). There were no significant differences observed between the group and time on any other dimension of the IMI (*P*>.05) (Figure 4).

Exercise adherence was measured using the child’s logbook. All participants performed 100% of the exercise as per each week’s exercise protocol. Furthermore, there were no reports of harm or injury occurring during training in either the AVG group or the CG.

## Discussion

### Principal Findings

The major finding of this study provides evidence that a 4-week AVG home-based exercise training program can have a positive and significant therapeutic effect on children with CAI as determined by improving static balance, foot and ankle ability, and key measures of intrinsic motivation (ie, interest/enjoyment, pressure/tension, and value/usefulness).

For balance control, after 4 weeks of the training program, the AVG group could perform the SLST with EO on a stable surface as well as on a foam surface better than those who performed the traditional CAI program. When playing video games, children learn how to act and respond to the game scenario promptly, which is considered to enhance sensorimotor learning after training [43]. Moreover, the improvement of balance following the AVG training could be related to the movement strategies inherent in the games. The games provide visual feedback via a variety of visual-perceptual processing challenges and prompt lower-extremity muscle responses to control posture and balance. The results of this study support previous findings that have demonstrated the effectiveness of using visual feedback tools as a therapeutic training modality [28,44-46]. For example, Kim and Heo [44] reported the benefits of 4 weeks of balance exercise using the Wii Fit Plus program (30 minutes, 3 times a week) for improving static balance in people with functional ankle instability. Moreover, Fitzgerald et al [28] found that a therapeutic exergaming system showed an improvement in postural stability when compared to a group doing similar balance training without the game system. Therefore, this study offers further support for using an AVG visual feedback training program as an effective modality for improving the balance of children with CAI.

The results of this study showed no significant difference in the subtest of the BOT-2 for functional balance, measured by walking forward on a line, and for leg muscle strength, measured by a standing long jump, in the AVG group and the CG after 4 weeks of training. This might be caused by either the movement patterns of the AVGs (moving feet to the left or right) and the traditional CAI program not being potent enough or the period of the exercise program (4 weeks) being too short to improve functional balance and lower-extremity strength.

This study revealed that foot and ankle ability measured by the FAAM-ADL in the AVG group improved after the 4-week training, and interestingly, it was greater than the CG. This result is congruent with the results of Punt et al [47], who found an improvement in foot and ankle ability during a 6-week follow-up of users of the Wii Fit game (twice per week, for 30 minutes per session) in lateral ankle sprain patients. Perceived instability of the foot and ankle has been reported to be associated with decreased health-related quality of life in individuals with CAI [43]. Thus, the exercise program with AVG could be used as exercise therapy to increase foot and ankle ability and might provide a better quality of life in children with CAI.

The participants’ subjective perception of the program was assessed every week. Notably, in week 2, the perception of interest/enjoyment was increased in the AVG group, whereas the perception of value/usefulness was decreased in the CG. Interestingly, the perception of pressure/tension was increased in the CG and greater than the AVG group at week 4. These results are consistent with previous findings [28,45,48] that reported that a video game–based rehabilitation approach could provide a greater intrinsic motivation level to engage in the activity for a longer period of time when compared to a group doing similar balance training. These results might explain that increased interest and enjoyment may have occurred due to the achievable challenges provided by the games (ie, Catching Fish and Russian Block) and the subsequent feelings of achievement when each game level was completed. By contrast, in the traditional CAI program, there was no feedback mechanism during the exercises. The participants simply had to count the number of repetitions and sets performed, and this might have contributed to the reduction in value/usefulness levels, as well as the pressure/tension level increasing during the program. It is noteworthy that the results of this study illustrated that effort/importance slightly declined after 2 weeks and was still declining in week 4. It may be possible that the children became accustomed to the exercise program, as they had to repeat the same exercise again and again to achieve the aim of the intervention. This result is consistent with the findings of previous research that reported nonadherence and lack of motivation in long-duration training in home-based therapeutic practices [15,16].

Furthermore, the protocol and training intervention using the AVG program has been shown to be feasible, as indicated by the retention and adherence of participants. The safety of the program is evidenced by the lack of adverse events reported. This suggests that the design and progression of the training had an appropriate intensity and the interactive video game did not present any harm. Therefore, the above results suggest that AVG programs provide an engaging and interactive environment that results in less fatigue, more relaxation, and enhanced adherence throughout the rehabilitation period.

### Limitations

There are some limitations to this study. First, observations were not obtained in a controlled laboratory environment. However, the researchers gave instructions, set up the exercise environment at the participants’ homes, and made telephone calls to give reminders to the participants. Second, the relatively small sample size may not be representative of the characteristics of the broader population of children with CAI. Third, follow-up measurements are required so that the longitudinal effects of the AVG program can be better understood. Lastly, a 4-week program is a relatively short intervention period. Thus, further studies would appear warranted to determine the effects of a longer-duration AVG training program.

### Conclusions

This study found that children with CAI showed signiﬁcant improvements in single-leg standing, foot, and ankle ability measured by the FAAM-ADL and intrinsic motivation in the interest/enjoyment, pressure/tension, and value/usefulness dimensions after following a home-based exercise program with AVGs. Furthermore, it was discovered that playing video games while exercising comprised a visuomotor feedback task that was lacking in the therapeutic exercise program. Thus, visual feedback while exercising with AVGs may be an effective way to improve balance ability, especially in the EO condition. This highlights that AVGs focused on balance exercises can be beneficial and may be an effective approach for improving balance and enhancing positive motivation in children with CAI who require long-term rehabilitation sessions.

## Supplementary material

10.2196/51073 Checklist 1 CONSORT-EHEALTH checklist (V 1.6.1).

## Abbreviations

ADL: activity of daily life
AVG: active video game
BOT-2: Bruininks-Oseretsky Test of Motor Proficiency, second edition
CAI: chronic ankle instability
CAITY: Cumberland Ankle Instability Tool-Youth
CG: control group
CONSORT: Consolidated Standards of Reporting Trials
EC: eyes closed
EO: eyes open
FAAM: Foot and Ankle Ability Measure
ICC: intraclass correlation coefficient
IMI: Intrinsic Motivation Inventory
IML: Intrinsic Motivation Level
SEBT: Star Excursion Balance Test
SLST: single leg stance test
